# Addressing mental health problems among persons without stable housing in the context of the COVID-19 pandemic: study protocol for a randomised trial. RESPOND – France

**DOI:** 10.1186/s12889-023-17238-7

**Published:** 2023-11-17

**Authors:** Maria Melchior, Natasha Figueiredo, Aurélia Roversi, Alexandre Dubanchet, Eric Bui, Julian Vadell-Martínez, Corrado Barbui, Marianna Purgato, José Luis Ayuso-Mateos, Roberto Mediavilla, David McDaid, A-La Park, Papoula Petri-Romão, Raffael Kalisch, Pablo Nicaise, Vincent Lorant, Marit Sijbrandij, Anke B. Witteveen, Richard Bryant, Mireia Felez, James Underhill, Giulia Pollice, Andrea Tortelli

**Affiliations:** 1 Sorbonne Université, INSERM, Institut Pierre Louis d’Epidémiologie et de Santé Publique, (IPLESP), Equipe de Recherche en Epidémiologie Sociale, Paris, F75012 France; 2 CHU Caen, & Normandie Univ, UNICAEN, INSERM, U1237, PhIND “Physiopathology and Imaging of Neurological Disorders”, NeuroPresage Team, Cyceron, Caen, 14000 France; 3 Department of Neuroscience, Biomedicine, and Movement Sciences, Section of Psychiatry, WHO Collaborating Centre for Research and Training in Mental Health and Service Evaluation, University of Verona, Verona, Italy; 4 Department of Psychiatry, Universidad Autónoma de Madrid (UAM), Madrid, Spain; 5 Instituto de Investigación Sanitaria del Hospital Universitario La Princesa, Madrid, Spain; 6 Centro de Investigación Biomédica en Red de Salud Mental (CIBERSAM), Instituto de Salud Carlos III, Madrid, Spain; 7 Care Policy and Evaluation Centre, Department of Health Policy, London School of Economics and Political Science, London, UK; 8 Leibniz Institute for Resilience Research (LIR), Mainz, Germany; 9 Neuroimaging Center (NIC), Focus Program Translational Neuroscience (FTN), Johannes Gutenberg University Medical Center, Mainz, Germany; 10 Institute of Health and Society (IRSS), Université Catholique de Louvain, Brussels, Belgium; 11 Clinical, Neuro and Developmental Psychology, WHO Collaborating Centre for Research and Dissemination of Psychological Interventions, Amsterdam Public Health Institute, VU University, Amsterdam, the Netherlands; 12 School of Psychology, University of New South Wales, Sydney, Australia; 13 Institut de recerca Sant Joan de Déu, Dr. Antoni Pujadas, Sant Boi de Llobregat (Barcelona), 4208830 Spain; 14 Brighton, UK; 15 GHU Paris, Psychiatrie & Neurosciences – Pôle Psychiatrie Précarité, Paris, France

**Keywords:** Randomized controlled trial, Psychological distress, COVID-19, Housing instability, Migrant, Doing what matters in times of stress, Problem management plus, Economic evaluation, France

## Abstract

**Background:**

The COVID-19 pandemic has had an impact on population-wide mental health and well-being. Although people experiencing socioeconomic disadvantage may be especially vulnerable, they experience barriers in accessing mental health care. To overcome these barriers, the World Health Organization (WHO) designed two scalable psychosocial interventions, namely the web-based Doing What Matters in Times of Stress (DWM) and the face-to-face Problem Management Plus (PM+), to help people manage stressful situations. Our study aims to test the effectiveness of a stepped-care program using DWM and PM + among individuals experiencing unstable housing in France – a majority of whom are migrant or have sought asylum.

**Methods:**

This is a randomised controlled trial to evaluate the effectiveness and cost effectiveness of a stepped-care program using DWM and PM + among persons with psychological distress and experiencing unstable housing, in comparison to enhanced care as usual (eCAU). Participants (N = 210) will be randomised to two parallel groups: eCAU or eCAU plus the stepped-care program. The main study outcomes are symptoms of depression and anxiety measured using the Patient Health Questionnaire Anxiety and Depression Scale (PHQ-ADS).

**Discussion:**

This randomised controlled trial will contribute to a better understanding of effective community-based scalable strategies that can help address the mental health needs of persons experiencing socioeconomic disadvantage, whose needs are high yet who frequently have limited access to mental health care services.

**Trial registration:**

this randomised trial has been registered at ClinicalTrials.gov under the number NCT05033210.

**Supplementary Information:**

The online version contains supplementary material available at 10.1186/s12889-023-17238-7.

## Background

The COVID-19 pandemic has contributed to a worldwide increase in the prevalence of psychological distress and symptoms of anxiety and depression, [1] although this has recently been a matter of some debate, in part due to heterogeneity in findings across different stages of the sanitary crisis [2]. While in the general population the increase in mental health problems was observed in 2020 and appears to have decreased afterwards, this may not be the case in marginalized groups. In particular, research has highlighted a high prevalence of mental health problems among individuals experiencing socioeconomic disadvantage compared to the general population [3, 4]. Mental health care systems are thus facing difficulties in addressing this growing demand, which may result in delays or unmet needs, increasing existing social inequalities in mental health and health care access [5].

In France in recent years, and more particularly in the Paris area, there has been an increase in the number of people who are homeless, sleeping rough or experience unstable housing conditions, among whom a large proportion are recent migrants and asylum seekers due in part to changes in migratory patterns and asylum regulations which contribute to newcomers’ social vulnerability [6]. In addition to social disadvantage, this group often experiences violence, discrimination, and isolation, as well as cultural and language barriers before, during and after migration, [7] and should have priority in terms of prevention of health conditions and treatment. However, this group experiences widely documented unmet needs in terms of mental health care. The COVID-19 pandemic has resulted in increased difficulties in accessing mental health care at large and in marginalized populations, calling for the evaluation of new scalable psychosocial interventions.

To make mental health care accessible, it is necessary to adapt it. For that purpose, the World Health Organization (WHO) has developed a number of scalable psychological interventions based on Acceptance and Commitment Therapy (ACT) including Doing What Matters in Times of Stress (DWM) [8] and Problem Management Plus (PM+) [9]. DWM is a self-help stress management guide that includes pre-recorded audio exercises and illustrations, introducing participants to five different strategies to relieve stress (e.g. mindfulness exercises) and has been shown to reduce psychological distress across multiple trials [10, 11]. To be widely accessible, DWM has been adapted for individual use as a mobile-supported website with distance support by a trained person spread over the course of 5 weeks. Self-Help + (SH+), is an intervention based on the DMW manual. The PM + intervention is based on principles of Cognitive Behavioural Therapy (CBT) and Problem-Solving Therapy and can be delivered by non-specialists in situations where mental health care is not accessible. PM + has four core features: it is brief (five sessions); delivered by non-specialist helpers; transdiagnostic (it addresses depression, anxiety, post-traumatic stress disorder, psychological distress); it was originally designed for individuals living in communities with limited mental health care resources but is easily adaptable to different populations, cultures and languages. Organised in 5 sessions, PM + can be delivered individually or in groups of adults experiencing distress to help them develop problem management strategies. Both individual and group formats of PM + have been shown to reduce anxiety and depression [12, 13].

These interventions do not replace specialised mental health care for people with severe psychiatric disorders, but contribute to reducing psychological distress and decrease later risk of mental disorders among persons with mild to moderate manifestations.

The main aim of the present randomised controlled trial (RCT) is to evaluate the effectiveness of the stepped-care program combining DWM and PM + to reduce symptoms of depression and anxiety amongst adults experiencing unstable housing in comparison to enhanced care-as-usual (eCAU). The secondary objectives are to evaluate:*(a)* the effect of the intervention on participants’ quality of life and resilience; *(b)* the moderation of the effect of the intervention by participants’ socio-demographic characteristics, living conditions, and experiences of trauma (i.e. individuals who have lower levels of education, less stable housing situation, and higher levels of trauma are expected to benefit less from the psychosocial program being tested);*(c)* the resource impacts and cost-effectiveness of DWM and PM + interventions. In France, a large proportion of individuals experiencing unstable housing conditions and sheltered in collective accommodation facilities are migrants [6], therefore the DWM and PM + protocols were professionally translated and back translated by study helpers who are native speakers and trained translators to languages most frequently spoken by recent asylum seekers and migrants to France (Dari and Pashto); we also used the existing Arabic translation provided by the WHO [14]. The study being conducted under the supervision of a psychiatrists (AT), the validity of the tools used to screen for psychological distress can be validated after the trial. PM + was previously used in studies conducted across different parts of the world (for instance in Pakistan and among Syrian refugees to Jordan) [12, 15]. Prior to the implementation of this randomised controlled trial, we conducted a pilot study in 2019–2020 to test the feasibility of recruiting participants for this intervention among people who do not speak French and reside in temporary accommodation. The pilot study allowed us to make a number of adjustments to study recruitment procedures (for instance the selection of participating accommodation centres) and research questionnaires, as well as estimate attrition. The presentRCT is part of the Horizon 2020 cross-national RESPOND project (Australia, Belgium, France, Germany, Italy, Netherlands, Spain, Sweden and the United Kingdom) aiming to test effective ways of addressing population mental health needs following the COVID-19 pandemic [16, 17]. This protocol was not peer-reviewed as part of the funding process.

## Methods: participants, intervention and outcomes

### Study setting

This study is set in the Paris and Caen regions in France, among people who experience unstable housing conditions (that is individuals who are homeless and are sheltered in temporary accommodation, by other people, or have no regular shelter) recruited via temporary accommodation centres as well as ambulatory care for people who experience social disadvantage and/or are migrant. The Paris region traditionally attracts new migrants because it concentrates economic resources. As a result, it a region where many infrastructures serve migrants – particularly those experiencing social disadvantage, making recruitment easier. As for Caen, it has also become an area concentrating migrants, due to its proximity to the UK. Moreover, one of the study’s collaborators (Dr. Bui) is based in Caen. We acknowledge that the selection of study locations may be a limitation of the protocol.

### Eligibility criteria

Individuals will be able to participate in the trial if they are 18 years of age or older, have elevated levels of psychological distress as assessed with the Kessler-10 scale (K10 score ≥ 15.9) [18], speak one of the study languages (Arabic, Dari, French, Pashto), find themselves without stable housing and are willing to participate.

Exclusion criteria include having an acute medical or psychiatric condition requiring urgent medical attention (e.g. hospitalization) as ascertained by the consultant psychiatrist involved in the trial (Dr. AT), a high risk of suicide, moderate/severe cognitive impairment (e.g. severe intellectual disability or dementia), being under legal protection (guardianship, curatorship, safeguard of justice), a change in the level of psychotropic treatment in the two months preceding the trial (as ascertained in the baseline study questionnaire at T1), and refusal to participate.

### Interventions

**Fig. 1:**
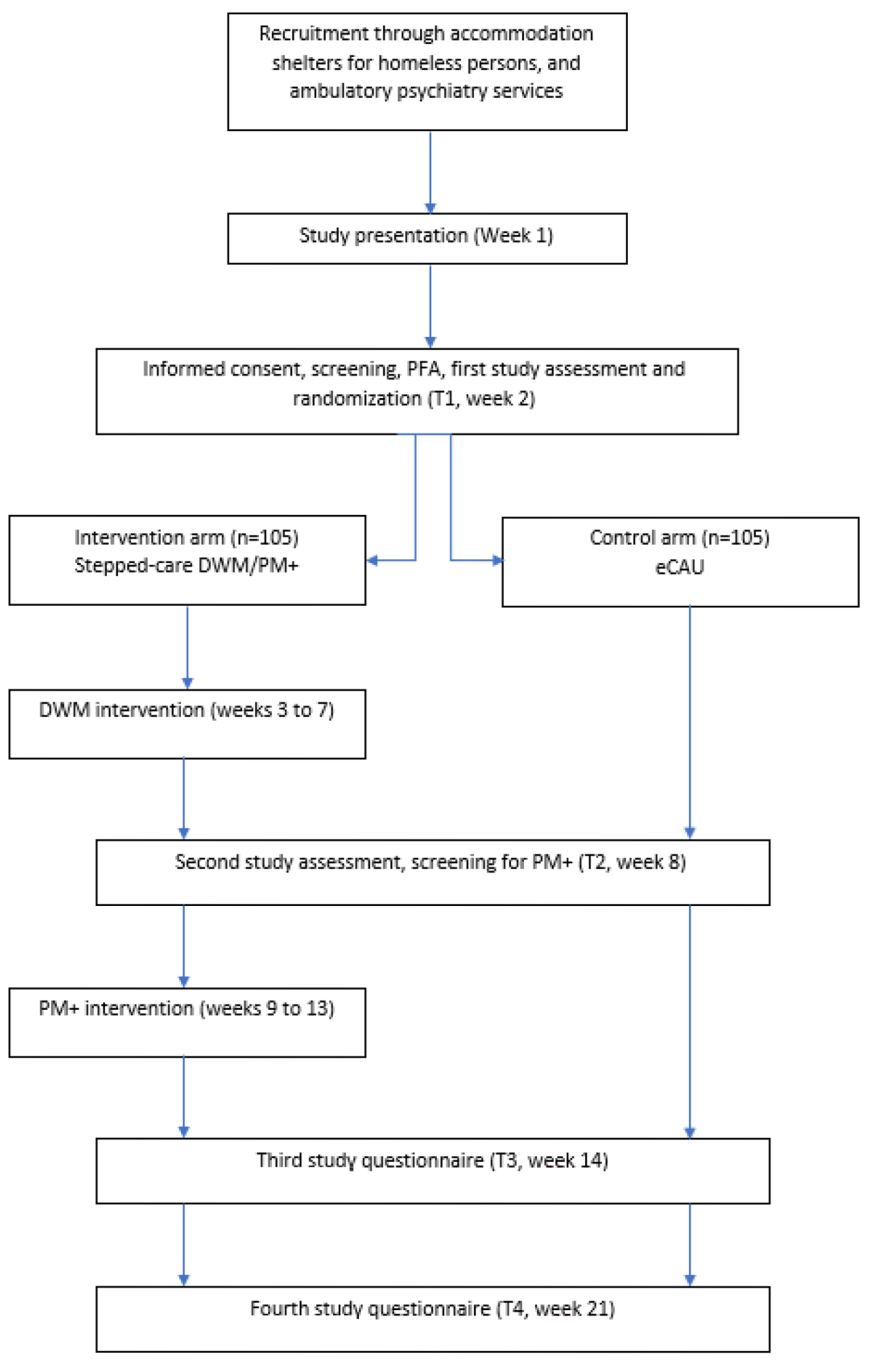
Framework of the RESPOND trial – France

This is a randomized controlled study, comparing a stepped-care intervention to enhanced Care as Usual (all participants will receive Psychological First Aid, PFA, prior to treatment allocation). All study assessments and interventions are described in Fig. 1. Participants randomly allocated to the intervention arm through the electronic platform used to collect study data will first receive the DWM intervention for 5 weeks (weeks 2 to 6). At the end of the DWM intervention, participants will complete the first follow-up questionnaire (T2 - week 7), which includes the K10 scale to evaluate whether they still experience high levels of psychological distress (K10 score ≥ 15.9). If so, they will receive the PM + intervention for the next 5 weeks (weeks 8 to 12) and then answer the second follow up questionnaire (T3 - week 13). Otherwise, they will skip the PM + intervention and only complete a follow-up questionnaire at week 13. Two months later, participants will complete the last follow-up questionnaire (T4 - week 21).

Participants allocated to the control group will complete the study questionnaires (T1, T2, T3 and T4) at the same time points as the intervention group and will not receive the DWM/PM + stepped-care program. The control group consists of enhanced Care as Usual, as participants receive Psychological First Aid (PFA) in addition to standard treatment.

1. Psychological First Aid (PFA) is a support strategy that involves humane, supportive and practical help for individuals experiencing a serious collective crisis [19] delivered to all study participants in both trial arms. PFA consists of a conversation (approximately 30–45 min) which can be provided remotely (e.g. videoconferencing or telephone). This conversation has various themes; non-intruding practical care and support, description of mental health needs and concerns, information about ways of addressing individuals’ basic needs, provision of comfort and help staying calm, help connecting to services and social support, as well as protection of individuals from further harm [20].
2. Doing What Matters in times of stress (DWM) is a self-help stress management guide based on the principles of Acceptance and Commitment Therapy (ACT). DWM includes five sections, each of which focuses on a specific skill (Grounding; Unhooking; Acting on your values; Being kind; Making room). In this study, DWM will be delivered online, via a web-based program designed to be used on a smartphone with the assistance of a helper. Helper support will be flexible, and on average occur once a week for approximately 15–20 min. Every week over the course of five weeks, a new module will be unlocked.
3. Problem Management + (PM+) is a brief, psychological intervention based on cognitive behavioural therapy (CBT) techniques that are empirically supported, formally recommended and developed by the WHO. The manual involves the following empirically supported elements: problem solving, stress management, behavioural activation, and accessing social support. In 60-minute sessions, participants talk to trained non-professional helpers. PM + sessions will be conducted face-to-face by a helper, also over a five-week period at a rate of one session per week.

The cultural acceptability of DMW and PM + among potential study participants and social workers attending to their needs was verified in qualitative research prior to study inception.

For both DWM and PM+, manuals can be accessed online [8, 9].

### Study outcomes

Table 1 shows all the measures collected across study questionnaires (from T1 to T4).

The primary study outcome is the Patient Health Questionnaire-Anxiety and Depression Score (PHQ-ADS), a sum of scores on the Patient Health Questionnaire-9 (PHQ-9) and the Generalized Anxiety Disorder − 7 (GAD-7), two scales evaluating respectively symptoms of depression and of generalised anxiety disorder [21, 22]. The PHQ-9 includes 9 items and the GAD-7 comprises 7 items, the combined score ranging from 0 to 48. Elevated scores indicate a higher level of symptoms of depression and anxiety. Two validation studies of the PHQ-ADS among patients with chronic pain and cancers have been summarized and published [22]. This score has strong internal reliability (Cronbach’s alpha of 0.8 and 0.9), strong convergent validity, sufficient uni-dimensionality and is sensitive to change.

As secondary study outcomes, we will use the PHQ-9 alone [23], the GAD-7 alone [24], as well as other measures of participants’ mental health: the Patient Checklist-5 (PCL-5) assessing symptoms of Post-Traumatic Stress Disorder [25], Psychotic Symptoms assessed using the MINI questionnaire [26] and quality of life assessed using the EQ-5D-5 L [27]. Additionally, we will assess outcome-based resilience, which is operationalised as the individual deviation from the sample-based normative stressor reactivity, that is a difference between individual-level observed and sample-based predicted values of psychological distress considering a certain number of stressors. Resilience will be calculated with a measure of stressor exposure which has been adapted from the Mainz Inventory of Microstressors(MIMIS) and the PHQ-9 and GAD-7 [28].

Additionally, baseline and follow-up study questionnaires will ascertain participants’ sociodemographic characteristics (sex, age, nationality, educational level, income, etc.), as well as COVID-19-related stressors (that is 11 items relative to worries regarding COVID-19 infection and possible stigma due to contamination, as well as loss of income, employment or basic life resources due to COVID-19 and possible administrative complications related to the sanitary crisis and its aftermath). Furthermore, participants are asked to report on their appraisal style using the Perceived Positive Appraisal Style Scale, content-focused version (PASSc) [28]. Moreover participants’ stress levels will be assessed using the Brief Trauma Questionnaire (BTQ) [29]. Finally, cost-effectiveness of the intervention will also be examined.

### Participant timeline

Each participant will be in the study for 21 weeks.

### Sample size

Overall, 210 participants will be included. Based on previous studies of PM+ [30, 31], we aim to detect a small to medium effect size corresponding to a Cohen’s d = 0.4 in the intervention group at 2 months, as measured with the PHQ-ADS and as compared to the control group. A calculation of statistical power for a repeated measures study design (mixed regression model), given incomplete participation in each of the planned assessments, suggests a minimum sample size of n = 73 per group (power = 0.80, alpha = 0, 05, bilateral, rho = 0.9). Considering an attrition rate of 30%, estimated based on a prior pilot study and past evaluations of PM+, we calculated a total number of 210 participants needed (105 in each group). If needed, missing data on study covariates will be handled using standard multiple imputation techniques.

### Recruitment

Participants are recruited directly in temporary accommodation centres as well as ambulatory care for people experiencing social disadvantage and/or who are migrant, by trained helpers who are in contact with each site’s coordinator and regularly visit to list potential participants and contact them. Recruitment and allocation started in March 2022 and is should be completed by the end of October, 2023. The first post-allocation took place in July 2022 and the last one will take place in February 2024, study close-out should occur in February 2024. To enhance retention and reduce study drop-out, participants are compensated 3*20 euros (at T1, T3 and T4). We also cover their travel expenses if they are interviewed on research premises rather than at the accommodation centre where they live.

### Adverse events

Any undesirable experience occurring during the study, whether or not considered related to the trial procedure or the stepped-care DWM/PM + program, will be considered an Adverse Event. All adverse events reported spontaneously by study participants or observed by the investigators will be recorded and reported to the promoting agency (ANRS-MIE).

## Methods : assignment of intervention

To evaluate the effectiveness and cost effectiveness of the DMW/PM + stepped-care program, we will conduct an RCT comparing the DMW/PM + stepped-care programme to enhanced care-as-usual (n = 105 each), delivered after all participants receive psychological first aid (PFA). After verification of inclusion criteria, participants will be randomly allocated to the intervention or eCAU based on a 1:1 basis, via the Castor Electronic Data Capture web-based software, also used to collect study data. This electronic tool applies a variable block randomization method, randomly permuting blocks of unequal size. The site investigators will be blind to the block size and will not have access to the randomization list.

Blinding of participants and data collectors to the intervention will not be possible due to the nature of the intervention being evaluated.

## Methods : data collection, management, analysis

A total of 210 participants will be recruited in two locations: Paris and Caen (Normandy, North Western France). In Paris, recruitment activities will be conducted via mental health care ambulatory services dedicated to migrants who experience social disadvantage (Precarity Unit of the GHU Paris, the largest psychiatric hospital in Paris), and accommodation centres for homeless people and asylum seekers. In Caen, participants will be recruited from accommodation centres for the homeless.

Potential participants will be informed about the study and given one week to decide whether they want to enrol. Participants who accept to enrol in the study and sign the informed consent form will complete the study screening questionnaire, which includes the K10 scale screening for psychological distress, as well as measures of sociodemographic and health characteristics. Participants who meet all inclusion criteria will complete the first study questionnaire and will be randomly allocated to one of the two study groups: intervention (PFA, DWM and PM + and CAU) or eCAU (PFA and CAU) (T1 - week 1).

Five helpers will be recruited to implement PFA, DWM and PM + among study participants. All helpers will be specially trained by a Master trainer and supervised weekly by a clinical psychologist throughout the study.

Prior to the recruitment of the first study participant, qualitative research activities were implemented to better understand the perceptions and expectations of eligible individuals. Initial qualitative interviews were conducted with 22 potential study participants as well as 26 professionals working in temporary accommodation centres, who are key in identifying and referring potential study participants. These qualitative interviews made it possible to adapt the research tools (for example the wording used in DMW and research questionnaires), as well as assess the acceptability of the proposed research protocol. At the end of the project, qualitative interviews with study participants as well as social workers and other support staff, will collect participants’ feedback and help gain information on ways of disseminating the program broadly.

**Table 1:** Measures assessed in the RESPOND Randomised Trial France

Measures	Screening (T1)	Follow-up at 7 weeks (T2)	Follow-up at 13 weeks (T3)		Follow-up at 21 weeks (T4)	
K10 (psychological distress)^16^	x	x				
Suicidal risk	x					
Suicidal screening step by step^36^	x	x	x		x	
PHQ-9 (symptoms of depression)^21–23^	x	x	x		x	
GAD-7 (symptoms of generalized anxiety disorder)^24–27^	x	x	x		x	
PCL-5 (symptoms of post-traumatic stress disorder)^28–30^	x	x	x		x	
PSYCHLOPS (Psychological Outcome Profiles)^37^	x	x	x		x	
Stress reactivity scores	x	x	x		x	
PASSc (STRENGTHS (Syrian REfuGees MeNTal HealTH Care Systems)^38^	x	x	x		x	
EQ-5D-5 L (quality of life)^32^	x	x	x		x	
MINI (psychotic symptoms)^31^	x	x	x		x	
CSRI (Client Service Receipt Inventory)^39^	x	x	x		x	
Sociodemographics	x	x	x		x	
BTQ (Brief trauma test)^40^	x		x		x	
Impact of COVID-19 on daily life activities	x	x	x		x	
Satisfaction with DWM^12^		x				
Satisfaction with PM + ^13^			x			

### Data collection methods

All study questionnaires (from T0 to T3) will be administered by a member of the research team or by a trained helper (Table 1). Face-to-face data collection will be prioritised but video or telephone meetings will be allowed if needed. Questionnaire administration and randomisation will be implemented using CASTOR EDC, an electronic CRF (eCRF). Table 2 describes the SPIRIT diagram for the schedule of enrolment, intervention and assessment.

**Table 2:** SPIRIT diagram for the schedule of enrolment, intervention and assessment in the RESPOND Randomised Trial France

	Study period						
	Enrolment	Allocation	DMW	Post allocation			Close-out
Timepoint	T0	T1		T2 (Week 7)	PM+	T3 (Week 13)	T4 (Week 21)
Enrolment	x						
*Information about the study*	x						
*Eligibility screen*		x					
*Informed consent*		x					
Allocation		x					
Interventions		x					
*Intervention group*		x					
*Control group*		x					
Assessments		x		x		x	x

### Data management

Quantitative data obtained from study questionnaires being collected on an eCRF will be automatically stored electronically. Each participant will be identified with a unique numerical identifier, all personal data used to contact participants being stored separately and securely. In compliance with French regulations, all data will be stored for two years after the end of the trial. In case of adverse events, information will be systematically collected, forwarded to the promoting agency and if necessary participation will be stopped. The trial will be audited after completion.

### Statistical methods

The data will be analysed using: *(a)* an intention-to-treat (ITT) analysis, which will include all participants recruited for the study (n = 210); *(b)* an analysis among participants who complete the program (per protocol - PP). The statistical analyses will be masked, i.e. the trial statistician will be blind to the group allocation until the analysis is complete. In addition, the trial statistician will not be involved in participant eligibility determination, intervention administration, outcome measurement, or data entry.

To estimate the effect of the intervention, a linear mixed model will be used. The intervention will be included as a fixed effect, the subject as the random effect, the baseline score of the primary outcome as a covariate. A covariate-adjusted linear mixed model of the primary endpoint will also be implemented adding covariates pre-specified at study baseline (gender, age, education, COVID-19-related events, and symptom severity, as well as pre-existing levels of depression and anxiety). The primary analysis will assess treatment effect on the average PHQ-ADS score at each time-point in the ITT population. The main conclusion of the trial will be drawn from this ITT analysis (i.e., the effect on PHQ-ADS score at the 2-month follow-up). In addition to the main analysis, secondary analyses will also be implemented. First, participants’ levels of psychological distress (PM+), symptoms of posttraumatic stress disorder (PCL-5), and quality of life (EQ-5D-5 L) will be evaluated at one-month post DWM (T2), as well as one week (T3) and two months post PM+ (T4). Second, changes in the number of cases of depression will be calculated in the PP sample using a logistic regression model.Third, the role of perceived positive appraisal style as measured by PASSc in relation to the first and secondary outcomes will be explored. Fourth, outcome-based resilience will be examined using mental health symptoms against stressor exposure. All statistical analyses will be implemented using Rstudio.

### Economic analysis

The primary outcome for the economic analysis will be the incremental costs per QALY gained between the intervention and comparator groups. The total costs of delivering interventions will be estimated and combined with data on changes in health service utilisation and time out of usual activity (from T1 to T4) obtained using a bespoke version of the Client Service Receipt Inventory (CSRI) [32], an instrument widely used for the collection of self-report service utilisation. Unit costs will be defined depending on the type of resource used. In addition to using the CSRI, we will collate information on the resources and costs of implementing the intervention, including initial and ongoing training/supervision. EQ-5D-3 L questionnaire responses from participants will be transformed into a preference-based index utility score using published French population tariffs [33] and QALYs will be calculated using the area under the curve approach. The economic analysis will be conducted from both the health care system and societal perspectives. Between-group comparisons of mean costs will be undertaken using appropriate statistical tests depending on the type and distribution of data. Univariate sensitivity analyses and non-parametric bootstrapping will be used to account for uncertainty in trial parameters and cost effectiveness planes will be generated. Cost-effectiveness acceptability curves will be constructed to indicate the likelihood that the intervention will be cost-effective at different levels of willingness to pay. The economic analysis will be conducted using STATA and SPSS.

## Ethics and dissemination

This research has obtained the approval of the Ethics Committee “*Île de France III*” on July 7 2021 (Comité de Protection des Personnes, 3858-I) and of the National body regulating data protection on February 2nd 2022 (Commission Nationale Informatique et Libertés, MLD /MFI/AR221796). The RESPOND Project - France respects the ethical principles stated in the reviewed version of the Helsinki Declaration of October 2013. The RESPOND trial is overseen by a Project Advisory Board, which includes independent experts and relevant stakeholders. Protocol amendments will be notified to the Comité de Protection des Personnes and authorization will be sought.

## Conclusion

Our study will test the effectiveness and cost-effectiveness of a stepped-care program including DWM and PM+, hypothesised to provide support to persons experiencing psychological distress and having difficulty accessing healthcare. Evidence suggests that stepped-care models are modestly effective [32], although there is a high heterogeneity of such models (number of steps, duration of steps, rules about stepping up) and their effects, implying the need for further research. Interestingly, there is also evidence that health care providers benefit from a switch from a matched care to a stepped-care approach [34]. Stepped-care interventions are both innovative and affordable since they can be administered face-to-face or remotely by non-professional helpers who receive special training. They could be particularly well-suited for people who experience social disadvantage, such as persons with an unstable housing situation and migrants, and face multiple barriers to access healthcare.

Overall, this study will contribute to identifying the impact of stepped-care programs on reducing psychological distress among marginalized populations who experience high levels of psychological distress yet have difficulty accessing mental health care. Results will contribute to providing effective community-based health care implementation strategies to scale-up the delivery and uptake of evidence-based mental health interventions in different contexts to address the specific needs. They will be disseminated to scientists, decisionmakers as well as study participants through dedicated publications and the Internet.

### Electronic supplementary material

Below is the link to the electronic supplementary material.

Supplementary Material 1

Supplementary Material 2

## Data Availability

Data collected in the RESPOND Project - France are available from the authors (maria.melchior@inserm.fr) upon reasonable request and with permission of INSERM|ANRS-MIE.
