# Supplementary material for: Addressing mental health problems among persons without stable housing in the context of the COVID-19 pandemic: study protocol for a randomised trial. RESPOND – France
Source: BMC Public Health. 2023 Nov 17;23:2275. doi: 10.1186/s12889-023-17238-7 (PMC10656934; doi:10.1186/s12889-023-17238-7)
Supplement: Supplementary file 2 — Supplementary Material 2 [file 12889_2023_17238_MOESM2_ESM.docx]

**Supplementary material**

**Supplementary Table1. Description of the Client Service Receipt Inventory Form (CSRI)**

I'm going to ask you about all the different contacts you've had with the health services over the last three months or since your arrival in France (if more recent).

| 1. | 1.Have you been in contact with any of these community health services in the last 3 months? | | | | | | | |
| --- | --- | --- | --- | --- | --- | --- | --- | --- |
|  | Health care providers | 0=No  1=Yes  If yes, ask the following questions | Number of contacts in past 3 months | Average cost of travel | Average time waiting for an appointment (in minutes) | Average travel time | Average time spent with healthcare provider | Average cost of visit |
| 1.1 | Community health worker/nurse |  |  |  |  |  |  |  |
| 1.3 | Primary care physician |  |  |  |  |  |  |  |
| 1.4 | Psychiatrist |  |  |  |  |  |  |  |
| 1.5 | Psychologist |  |  |  |  |  |  |  |
| 1.6 | Psychiatric nurse |  |  |  |  |  |  |  |
| 1.7 | Social worker |  |  |  |  |  |  |  |
| 1.8 | Physical therapist |  |  |  |  |  |  |  |
| 1.9 | Homebased care |  |  |  |  |  |  |  |
| 1.10 | Support group |  |  |  |  |  |  |  |
| 1.11 | Addiction center |  |  |  |  |  |  |  |
| 1.12 | Psychiatric emergency service |  |  |  |  |  |  |  |
| 1.13 | Other |  |  |  |  |  |  |  |

| 2 | Have you been hospitalized in the last 3 months ? | 0=No  1=Yes  If yes, ask the following questions | If yes: Planned =1  Non planned = 2 |
| --- | --- | --- | --- |
|  | Hospitalisation ward | Number of days of stay | Total cost covered by yourself |
| 2.1 | Mental health unit |  |  |
| 2.2 | Long-term psychiatric care |  |  |
| 2.3 | Other type of care |  |  |

| 3. | Have you attended hospital ambulatory care in the last 3 months ? | 0=No  1=Yes  If yes, ask the following questions |  |
| --- | --- | --- | --- |
|  | Hospital ward | Unit | Number of visits |
| 3.1 | Emergency service | Visit |  |
| 3.2 | Ambulatory psychiatric care | Visit |  |
| 3.3 | Other ambulatory care | Visit |  |

| 4. | In the last 3 months, have you taken medication for your mental health? If yes, ask the following questions | | | 0=No  1=Yes |  |
| --- | --- | --- | --- | --- | --- |
|  | If yes, what medication? | If yes, name/description of medication | Mode of acquisition  1=Paid for    2=Complementary | Dosage (if known) | Frequence of dosage  1=3 times per day  2 = 2 times per day ;  3 = One time per day  4 = Weekly  5 = Every other week  6 = Monthly  7 = Less than once a week |

| 5. | In the last 3 months, have you had access to traditional or unconventional health services ?  (0) No □  (1) Yes □  If yes, ask the following questions | | | | | | | |
| --- | --- | --- | --- | --- | --- | --- | --- | --- |
|  |  | Type/ Name of service | Number of contacts in last 3 months | Average cost of travel | Average time waiting for an appointment (in minutes) | Average travel time | Average time spent with healthcare provider | Average cost of visit |
| 5.1 |  |  |  |  |  |  |  |  |
| 5.2 |  |  |  |  |  |  |  |  |

| 6. | In the last 3 months, have you, your family or a friend, had to stop or reduce your usual work/activities because of your poor health? | 0= No | 1=Yes |
| --- | --- | --- | --- |

|  | *If yes*: | *Patient* | *Family / friend 1* | *Family / friend 2* |
| --- | --- | --- | --- | --- |
| 6.1 | Number of days in last 3 months |  |  |  |
| 6.2 | Type of work abandoned (1-3)  1 = unpaid housework (e.g. housewife)  2 = manual work (e.g. farm or factory worker)  3 = office/non-manual work (e.g. skilled worker, business, professional) |  |  |  |
| 6.3 | If relevant, income lost per day |  |  |  |

Additional question

| 7. | In the last 3 months, have you consulted a healthcare professional (formally or informally) outside France about your (mental) health problems? | 0= Non | 1=Oui |
| --- | --- | --- | --- |
| 7.1 | If yes, please describe |  | |
